# Are ambulatory blood pressure parameters associated more with central adiposity than with total adiposity? Results of the ELSA-Brasil study

**DOI:** 10.3389/fcvm.2023.1286726

**Published:** 2023-12-14

**Authors:** Ângela Maria Natal de Souza, Rosane Harter Griep, Helen Hermana Miranda Hermsdorff, Maria de Jesus Mendes da Fonseca, Leidjaira Lopes Juvanhol

**Affiliations:** ^1^ Department of Nutrition and Health, Federal University of Viçosa, Viçosa, Brazil; ^2^ Environment and Health Education Laboratory, Oswaldo Cruz Institute, Oswaldo Cruz Foundation, Rio de Janeiro, Brazil; ^3^ National School of Public Health, Oswaldo Cruz Foundation, Rio de Janeiro, Brazil

**Keywords:** adiposity, ambulatory blood pressure monitoring, nocturnal dipping, morning surge, variability

## Abstract

**Background:**

Worldwide obesity has a high prevalence, as well as carries a high risk of several chronic diseases, including hypertension. Studies of the association between obesity and ambulatory blood pressure (BP) are scarce and most use only body mass index (BMI) as indicator of adiposity. Thus, we aimed to examine for associations between total and central adiposity and ambulatory BP parameters (BP means and variability, nocturnal dipping and morning surge) among participants in the Brazilian Longitudinal Study of Adult Health (ELSA-Brasil).

**Methods:**

This cross-sectional study (2012–2014) used a subsample of participants (*n* = 812) of ELSA-Brasil who underwent 24-hour ambulatory BP monitoring to assess systolic and diastolic BP (SBP and DBP, respectively) over 24-hour periods and sub-periods. Indicators for total adiposity were BMI and body fat (BF) and, for central adiposity, waist circumference (WC) and waist-to-height ratio (WHR). Associations were tested using crude and adjusted gamma and logistic regression.

**Results:**

Overweight (BMI) and abdominal obesity (WC and WHR) associated positively with mean 24-hour (Coef = 2.71, 3.09 and 4.00, respectively), waking (Coef = 2.87, 3.26 and 4.16, respectively), and sleeping (Coef = 2.30, 2.74 and 3.50, respectively) SBP; mean DBP associated with high WHR in these three periods (Coef = 2.00, 2.10 and 1.68, respectively) and with WC in the waking period (Coef = 1.44). Overweight and abdominal obesity (WC and WHR) were positively associated with SBP variability over 24 h (Coef = 0.53, 0.45 and 0.49, respectively) and in sleep (Coef = 0.80, 0.74 and 0.59, respectively), and with DBP variability in 24 h (Coef = 0.64, 0.73 and 0.58, respectively), wakefulness (Coef = 0.50, 0.52 and 0.52, respectively) and sleep (Coef = 0.53, 0.45 and 0.49); excess BF associated positively with DBP variability over 24 h (Coef = 0.43) and in wakefulness (Coef = 0.38). Lastly, high WHR and excess BF were associated with higher odds of extreme dipping (OR = 1.03 for both), while high WC and WHR associated with higher odds of exacerbated diastolic morning surge (OR = 3.18 and 3.66, respectively).

**Conclusion:**

Indicators of adiposity were associated with the BP means and variability, nocturnal dipping and morning surge, with more substantial results for indicators of central adiposity that the others.

## 1. Introduction

Worldwide obesity has a high prevalence, as well as carries a high risk of several chronic diseases, including hypertension (HTN) (1), which is responsible for around 8.5 million global deaths from cardiovascular and kidney diseases (2). However, most of the available evidence on this relationship is based on casual blood pressure (BP) measurement (3, 4), while studies using data from ambulatory BP monitoring (ABPM) are scarce (5–7).

ABPM is in fact suitable for evaluating BP over 24 h and in subperiods of the day (5). This evaluation at specific moments in the day is of major clinical importance, particularly as regards sleeping BP, in view of its potential for predicting cardiovascular risk (8). Also, with data from ABPM, it is possible to evaluate markers relating to BP circadian rhythms, such as nocturnal dipping and morning surge, which cannot be assessed by casual BP measurement. Nocturnal dipping is a physiological phenomenon characterised by a drop in BP levels during sleep from their waking levels (9). When nocturnal dipping is absent or attenuated (a drop of less than 10%) (10), it is an important cardiovascular risk factor, even among individuals whose BP is within normal limits (11). Morning surge, the rise in BP levels in the morning on waking and starting activities (10), is also a cardiovascular risk factor when exacerbated (4). Furthermore, ABPM is a more accurate method for diagnosing hypertension and predicting target organ damage (12, 13). Finally, ABPM provides a greater number of measurements and a profile of BP behavior outside the office (14), allowing a more accurate assessment of antihypertensive therapy effectiveness over 24 h (10), as well as diagnosing white coat hypertension and masked hypertension (15).

In addition, most studies of the relation between obesity and BP based on ABPM data have used body mass index (BMI) to evaluate adiposity (5, 16). However, BMI has limitations as a measure, such as its not distinguishing between lean mass and fat mass and thus possibly overestimating percentage fat (17). Nor can it be used to assess body fat distribution, which is another limitation, given that accumulated fat in the abdominal region has been identified as an important cardiovascular risk factor (18). Accordingly, the literature recommends using BMI in combination with other indicators, such as waist circumference (WC) and waist-to-height ratio (WHR) to improve the assessment of body adiposity (19), especially considering that these indicators have proven better predictors of chronic noncommunicable diseases (20), such as HTN (21).

Thus, we aimed to examine for associations between total and central adiposity and ambulatory BP parameters (BP means and variability, nocturnal dipping and morning surge) among participants in the Brazilian Longitudinal Study of Adult Health (ELSA-Brasil).

## 2. Methods

### 2.1. Study design and population

This cross-sectional study used data from a subsample of ELSA-Brasil, a Brazilian multicentre cohort whose baseline (wave 1) was between 2008 and 2010 (*n* = 15,105). All civil servants from 35 to 74 years old from the participating institutions (Federal Universities of Bahia, Espírito Santo, Minas Gerais, São Paulo and Rio Grande do Sul and Fiocruz – RJ), whether active or retired, were considered eligible. The exclusion criteria were: intention to relinquish the post, pregnancy (temporary exclusion), impaired cognitive and communication capacity, and retirement residence outside the metropolitan area of the study centre. Other methodological details were described previously (22, 23).

The main objective of ELSA-Brasil was to investigate the incidence of chronic diseases (diabetes and cardiovascular diseases). Thus, the sample calculation considered a significance level of 5%, statistical power of 80%, a prevalence of exposure of 20% and relative risk equal to 2.0. This calculation resulted in 6,400 individuals. However, the final sample size was approximately 15,000 individuals, as specific gender analyzes and losses to follow-up were considered. More methodological details have been described previously (22).

**Figure 1:**
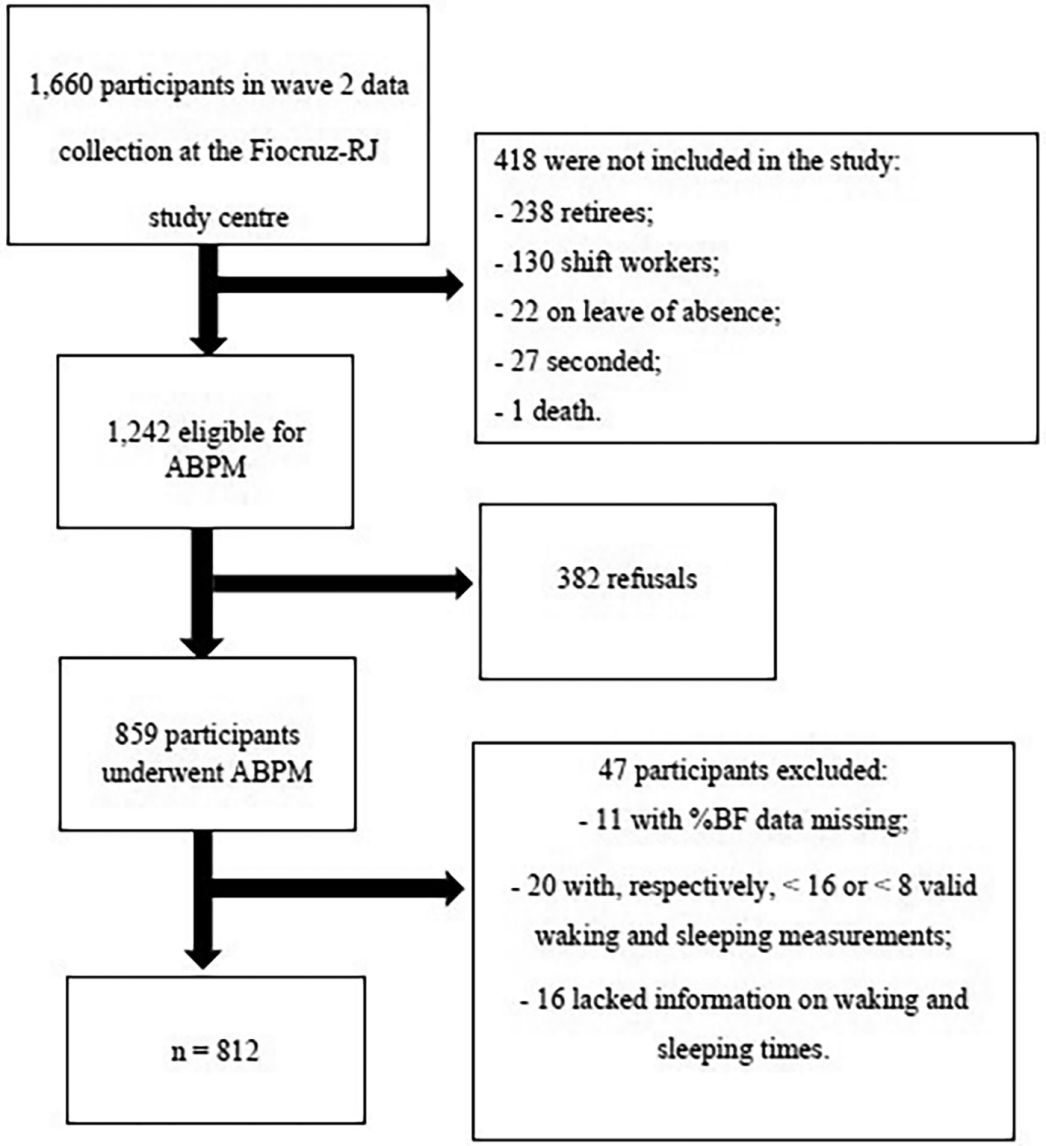
Flow diagram of study participant inclusion.

During wave 2 of ELSA-Brasil (2012–2014; *n* = 14,014), all eligible participants for ABPM from the Fiocruz-RJ study centre (active workers, non-shift workers and those not absent or seconded) were invited to undergo 24-hour ABPM in a subsequent visit to the participant's place of work. We excluded from this study those with ≤16 or ≤8 valid waking and sleeping BP measurements, respectively (13), those lacking information on waking and sleeping hours or missing data on the main study variables. The final sample consisted of 812 participants (Figure 1).

ELSA-Brasil was approved by Brazil's National Research Ethics Commission (Conep – No. 13065) and all participants signed a declaration of free, informed consent.

### 2.2. Study variables

#### 2.2.1. Blood pressure

A ABPM equipment (Spacelabs®, model 90,207), was fitted on each participant's non-dominant arm (so as not to hamper their daily activities), shortly after their arrival at the workplace. Cuff size was selected to suit the participant's arm circumference and the equipment was set to take measurements every 20 min between 6 a.m. and 11 p.m. and every 30 min between 11 p.m. and 6 a.m (24). Individuals were instructed to maintain a routine and keep diaries with periods of activities and medications and were asked to avoid performing physical leisure activities and drinking alcoholic beverages. From the measurements obtained by ABPM, mean systolic and diastolic BP (SBP and DBP, respectively) values were calculated for the 24 h and waking and sleeping subperiods of the day, as well as BP variability given by the standard deviation of BP in the same periods. Also calculated were nocturnal dipping [(waking BP - sleeping BP)/waking BP × 100], which was classified as normal (10%–20%); non-dipping (<10%); and extreme (>20%) (13). In this study, SBP dipping alone was considered (25). Lastly, morning surge was obtained as the difference between morning BP (the mean of values recorded in the two hours after waking) and the mean of values recorded in the two hours prior to waking, termed pre-awakening morning surge (26), and was classified as exacerbated when equal to or greater than the last distribution decile (≥23.95 and ≥21.2 mmHg for SBP and DBP, respectively) (16).

The criteria for HTN were: use of anti-hypertensive medicines or BP levels above the cut-off point for each period: ≥130/80 mmHg over 24 h, ≥135/85 mmHg waking and ≥120/70 mmHg sleeping (13, 27).

Casual BP was measured at the participant's workplace, prior to fitting the ABPM apparatus. Two BP measurements were taken at one-minute intervals with automatic oscillometric BP monitor (Omron Intellisense®, model 705CP), using a standardized protocol (28). Participants were classified as with HTN by casual measurement when SBP ≥ 140 mmHg or DBP ≥ 90 mmHg (29) or when using anti-hypertensive medicines.

#### 2.2.2. Indicators of adiposity

Anthropometric measurements were taken during wave 2 of ELSA-Brasil, with participants having fasted for 12 h. Height in centimetres was measured using a SECA-SE-216 stadiometer, to a precision of 0.1 cm, by standard technique (30). Weight was measured using platform scales (Toledo, São Bernardo do Campo, São Paulo, Brazil), to 50 g precision (31). BMI was calculated, and overweight was considered to exist at ≥25 kg/m^2^ and ≥28 kg/m^2^ for adults and older adults, respectively (32, 33).

WC was measured at the mid-point between the last rib and iliac crest, with the participant standing, and the following cut-off points were used: ≥90 cm for men and ≥80 cm for women (34). WHR was also calculated and considered high in either sex at ≥0.5 (35).

Bioelectrical impedance analysis was conducted using Inbody 230 equipment (BioSpace, Seoul, South Korea), to the manufacturer's recommendations. Men and women with percentage body fat (%BF) ≥25% and ≥32%, respectively, were classified as with excess BF (36).

#### 2.2.3. Covariables

Data on sociodemographic and behavioural characteristics were obtained by interview, during which a structured questionnaire was applied, at wave 2 of ELSA-Brasil. The following covariables were considered: age (complete years); sex (male and female); self-reported race/colour [black, white, *pardo* (mixed), and yellow and indigenous, with the latter two grouped as “Others”]; schooling (up to complete upper secondary, complete/incomplete university and postgraduate); binge drinking (Yes or No), considered to exist at intake of ≥5 doses of alcoholic beverage in a two-hour period more than once a month (37); and leisure-time physical activity, assessed by the International Physical Activity Questionnaire (IPAQ) and given as the weighted sum of physical activities per week in metabolic equivalent minutes (MET-minutes), which was categorised into weak, moderate and strong (38).

### 2.3. Statistical analyses

The sample was characterised by calculating medians (interquartile range) and absolute and relative frequencies for quantitative and qualitative variables, respectively. The Shapiro-Wilk test and graph-based analyses were used to evaluate the normality.

Associations between the indicators for adiposity and means and variability of 24-hour, waking and sleeping BP were evaluated using gamma regression, as appropriate for asymmetrical, strictly positive, continuous data (39). The identity link function was used. Multinomial logistic regression models were also used to investigate the association between indicators of adiposity and nocturnal dipping. Lastly, binary logistic regression was used to investigate the association between indicators of adiposity and morning surge. Crude models were estimated, considering each exposure variable individually (indicators of adiposity) and adjusted models were estimated for potential confounders defined on the basis of the literature and statistical exploration of the data (age, sex, race/colour, schooling, use of anti-hypertensives, physical activity and binge drinking). Also, in sensitivity analysis, the models were adjusted for variability, by the mean BP levels in each period (24-hour, waking and sleeping) and, for nocturnal dipping and morning surge, by 24-hour BP.

All analyses were performed to a 5% level of significance, using R (version 4.0.2).

## 3. Results

Participant characteristics according to sociodemographic and behavioural variables and indicators of adiposity are presented in Table 1.

**Table 1:** Characterisation of study population by sociodemographic and behavioural variables and indicators of adiposity.

Variable	*n* (%) or median (IQR)
Age (years)	51 (46–56)
Sex	
Female	406 (50.0)
Male	406 (50.0)
Race/colour^a^	
Black	101 (12.5)
*Pardo*	233 (28.9)
White	446 (55.3)
Others	27 (3.3)
Schooling	
Up to complete upper secondary	126 (15.5)
University incomplete/complete	109 (13.4)
Postgraduate	577 (71.1)
Level of physical activity	
Weak	585 (72.0)
Moderate	145 (17.9)
Strong	82 (10.1)
Binge drinking^b^	
Yes	677 (83.6)
No	133 (16.4)
Overweight (BMI)	
Yes	527 (64.9)
No	285 (35.1)
Abdominal obesity (WC)	
Yes	626 (77.1)
No	186 (22.9)
Abdominal obesity (WHR)	
Yes	658 (81.0)
No	154 (19.0)
Excess BF (%BF)	
Yes	594 (73.2)
No	218 (26.8)

BMI, body mass index; BF, body fat; WC, waist circumference; WHR, waist-to-height ratio.

Cut-off points: BMI ≥ 25 kg/m^2^ or ≥28 kg/m^2^ for adults and older adults, respectively; WHR ≥ 0.5; WC ≥ 90 cm for men and ≥80 cm for women; BF ≥ 25% in men and ≥32% in women.

ELSA-Brasil, 2012–2014 (*n* = 812).

^a^ 5 participants with missing data on race/colour.

^b^ 2 participants with missing data on binge drinking (exists at intake of ≥5 doses of alcoholic beverages in a two-hour period more than once a month).

The highest BP levels were observed in ambulatory BP during the waking period, followed by the 24-hour and casual BP levels; the lowest levels were observed during sleep. Use of anti-hypertensive medicines was reported by 30% of participants and more than 55% were classified as with HTN by ABPM. On casual measurement, prevalence of HTN was 39.4% (Table 2).

**Table 2:** Characterisation of the study population by BP levels.

Variable	*n* (%) or median (IQR)
24-hour SBP (mmHg)	123.5 (116.6–131.8)
Waking SBP (mmHg)	127.2 (120.6–135.9)
Sleeping SBP (mmHg)	111.1 (104.0–120.0)
24-hour DBP (mmHg)	78.1 (73.3–84.2)
Waking DBP (mmHg)	81.7 (76.7–87.5)
Sleeping DBP (mmHg)	67.1 (61.1–73.6)
Casual SBP (mmHg)	120.0 (111.0–130.5)
Casual DBP (mmHg)	76.5 (71.0–84.0)
24-hour HTN	
Yes	475 (58.5)
No	337 (41.5)
Waking HTN	
Yes	459 (56.5)
No	353 (43.5)
Sleeping HTN	
Yes	451 (55.5)
No	361 (44.5)
Casual HTN^a^	
Yes	316 (39.4)
No	487 (60.6)
Use of anti-hypertensive medicines	
Yes	245 (30.0)
No	567 (70.0)
Nocturnal dipping	
Normal	492 (57.6)
Non-dipping	256 (35.5)
Extreme	64 (7.9)
Exacerbated systolic morning surge^b^	
Yes	52 (10.2)
No	459 (89.2)
Exacerbated diastolic morning surge^b^	
Yes	52 (10.2)
No	459 (89.2)

BP, blood pressure; HTN, hypertension; IQR, interquartile range; DBP, diastolic blood pressure; SBP, systolic blood pressure.

Cut-off points: 24-hour HTN: ≥130/80 mmHg; Waking HTN: ≥135/85 mmHg; Sleeping HTN: ≥120/70 mmHg; Casual HTN: ≥140/90 mmHg; Nocturnal dipping: normal ≥10% and <20%, non-dipping <10% and extreme ≥20%; Morning surge: systolic ≥23.95 mmHg; diastolic ≥21.2 mmHg.

ELSA-Brasil, 2012–2014 (*n* = 812).

^a^ 9 participants with missing data for casual HTN.

^b^ 56 participants with missing data for morning surge.

In the gamma regression models for mean BP levels, overweight (by BMI) was associated with increases of 2.71 (95% CI = 1.10–4.30) mmHg in 24-hour SBP, 2.87 (95% CI = 1.23–4.50) mmHg in waking SBP and 2.30 (95% CI = 0.56–4.03) mmHg in sleeping SBP. Elevated WC was found to associate with increases in 24-h, waking and sleeping SBP and with an increase in waking DBP. Higher WHR values were also found to associate with SBP and DBP increases in 24-h, waking and sleeping measurements. Lastly, overweight and abdominal obesity (WC and WHR) associated positively with casual SBP and DBP, while excess BF associated only with the latter (Table 3).

**Table 3:** Coefficients and confidence interval for association between indicators of adiposity and mean BP.

Models	Overweight (BMI)	Abdominal obesity (WC)	Abdominal obesity (WHR)	Excess BF (%BF)
	Coeff. (95% CI)	Coeff. (95% CI)	Coeff. (95% CI)	Coeff. (95% CI)
24-hour SBP (mmHg)				
Crude model	4.03 (2.39–5.65)	4.23 (2.39–6.06)	5.78 (3.83–7.69)	−0.67 (−2.47–1.12)
Adjusted model^a^	2.71 (1.10–4.30)	3.09 (1.30–4.85)	4.00 (2.11–5.87)	−0.48 (−2.30–1.32)
Waking SBP (mmHg)				
Crude model	4.13 (2.48–5.77)	3.23 (1.40–5.04)	4.15 (2.22–6.07)	−0.55 (−2.38–1.26)
Adjusted model^a^	2.87 (1.23–4.50)	3.26 (1.44–5.07)	4.16 (2.23–6.07)	−0.41 (−2.25–1.44)
Sleeping SBP (mmHg)				
Crude model	3.61 (1.82–5.39)	4.09 (2.08–6.08)	5.58 (3.46–7.67)	−0.40 (−2.37–1.55)
Adjusted model^a^	2.30 (0.56–4.03)	2.74 (0.80–4.66)	3.50 (1.45–5.53)	−2.15 (−2.19–1.74)
24-hour DBP (mmHg)				
Crude model	1.67 (0.43–2.91)	1.54 (0.12–2.94)	3.06 (1.58–4.53)	−1.40 (−2.77 - −0.05)
Adjusted model^a^	0.53 (−0.69–1.75)	1.24 (−0.12–2.60)	2.00 (0.56–3.43)	−0.26 (−1.64–1.11)
Waking DBP (mmHg)				
Crude model	1.85 (0.58–3.12)	1.66 (0.21–3.09)	3.04 (1.51–4.54)	−1.29 (−2.70–0.09)
Adjusted model^a^	0.73 (−0.53–1.98)	1.44 (0.03–2.84)	2.10 (0.61–3.57)	−0.13 (−1.55–1.28)
Sleeping DBP (mmHg)				
Crude model	1.12 (−0.21–2.46)	1.33 (−0.18–2.83)	3.05 (1.46–4.61)	−1.14 (−2.61–0.30)
Adjusted model^a^	0.11 (−1.20–1.41)	0.77 (−0.69–2.22)	1.68 (0.13–3.20)	−0.22 (−1.70–1.25)
Casual SBP (mmHg)				
Crude model	7.24 (5.12–9.34)	6.78 (4.39–9.14)	8.12 (5.59–10.61)	−0.22 (−2.61–2.14)
Adjusted model^a^	5.34 (3.37–7.30)	4.91 (2.71–7.09)	5.06 (2.72–7.27)	0.38 (−1.89–2.62)
Casual DBP (mmHg)				
Crude model	5.14 (3.76–6.52)	4.52 (2.94–6.06)	5.58 (3.92–7.21)	1.11 (−0.44–2.65)
Adjusted model^a^	3.96 (2.55–5.34)	3.91 (2.37–5.44)	4.31 (2.67–5.92)	1.92 (0.34–3.49)

Coeff., coefficient; BF, body fat; CI, confidence interval; BMI, body mass index; BP, blood pressure; DBP, diastolic blood pressure; SBP, systolic blood pressure; WC, waist circumference; WHR, waist-to-height ratio.

Cut-off points: BMI ≥ 25 kg/m^2^ or ≥28 kg/m^2^ for adults and older adults, respectively; WHR ≥ 0.5; WC ≥ 90 cm for men and ≥80 cm for women; %BF ≥ 25% in men and ≥32% in women.

ELSA-Brasil, 2012–2014 (*n* = 812).

^a^ Model adjusted for age, sex, race/colour, schooling, physical activity, binge drinking and use of anti-hypertensives.

Overweight, elevated WC and WHR were also associated with increases in 24-hour and sleeping SBP variability. DBP variability was found to associate positively with increased values of all the indicators of adiposity evaluated, both over 24 h and in the subperiods, except between sleeping DBP variability and excess BF (Table 4).

**Table 4:** Coefficients and confidence interval for association between indicators of adiposity and BP variability.

Models	Overweight (BMI)	Abdominal obesity (WC)	Abdominal obesity (WHR)	Excess BF (%BF)
	Coeff. (95% CI)	Coeff. (95% CI)	Coeff. (95% CI)	Coeff. (95% CI)
24-hour SBP (mmHg)				
Crude model	0.51 (0.12–0.89)	0.59 (0.15–1.02)	0.60 (0.12–1.05)	0.55 (0.13–0.97)
Adjusted model^a^	0.53 (0.12–0.93)	0.45 (0.03–0.89)	0.49 (0.08–0.95)	0.27 (−0.17–0.71)
Waking SBP (mmHg)				
Crude model	0.27 (−0.07–0.61)	0.41 (0.02–0.78)	0.49 (0.08–0.89)	0.55 (0.14–0.97)
Adjusted model^a^	0.29 (−0.05–0.63)	0.14 (−0.24–0.51)	0.30 (−0.10–0.69)	0.23 (−0.14–0.61)
Sleeping SBP (mmHg)				
Crude model	0.87 (0.50–1.24)	0.87 (0.45–1.27)	0.77 (0.32–1.21)	0.41 (−0.01–0.81)
Adjusted model^a^	0.80 (0.42–1.18)	0.74 (0.31–1.14)	0.59 (0.13–1.02)	0.41 (−0.02–0.84)
24-hour DBP (mmHg)				
Crude model	0.65 (0.35–0.95)	0.69 (0.35–1.02)	0.51 (0.14–0.87)	0.43 (0.10–0.75)
Adjusted model^a^	0.64 (0.33–0.96)	0.73 (0.39–1.07)	0.58 (0.20–0.94)	0.43 (0.08–0.78)
Waking DBP (mmHg)				
Crude model	0.51 (0.24–0.77)	0.60 (0.30–0.90)	0.53 (0.20–0.84)	0.50 (0.21–0.79)
Adjusted model^a^	0.50 (0.21–0.77)	0.52 (0.21–0.83)	0.52 (0.19–0.84)	0.38 (0.06–0.68)
Sleeping DBP (mmHg)				
Crude model	0.80 (0.47–1.12)	0.88 (0.53–1.23)	0.74 (0.35–1.11)	0.50 (0.14–0.84)
Adjusted model^a^	0.53 (0.12–0.93)	0.45 (0.03–0.89)	0.49 (0.08–0.95)	0.27 (−0.17–0.71)

Coeff., coefficient; BF, body fat; CI, confidence interval; BMI, body mass index; BP, blood pressure; DBP, diastolic blood pressure; SBP, systolic blood pressure; WC, waist circumference; WHR, waist-to-height ratio.

Cut-off points: BMI ≥ 25 kg/m^2^ or ≥28 kg/m^2^ for adults and older adults, respectively; WHR ≥ 0.5; WC ≥ 90 cm for men and ≥80 cm for women; %BF ≥ 25% in men and ≥32% in women.

ELSA-Brasil, 2012–2014.

^a^ Model adjusted for age, sex, race/colour, schooling, physical activity, binge drinking and use of anti-hypertensives.

As regards associations between indicators of adiposity, nocturnal dipping and morning surge (Table 5), elevated WHR and excess BF were associated with higher odds of extreme nocturnal dipping. Abdominal obesity, whether evaluated by WC or WHR, associated with higher odds of exacerbated diastolic morning surge.

**Table 5:** Odds ratio and confidence intervals for the association between indicators of adiposity and nocturnal dipping and morning surge.

Models	Overweight (BMI)	Abdominal obesity (WC)	Abdominal obesity (WHR)	Excess BF (%BF)
	OR (95% CI)	OR (95% CI)	OR (95% CI)	OR (95% CI)
Non-dipping				
Crude model	1.01 (0.77–1.37)	1.19 (0.83–1.72)	1.23 (0.83–1.82)	1.21 (0.85–1.77
Adjusted model^a^	0.93 (0.67–1.29)	1.05 (0.72–1.53)	1.01 (0.68–1.51)	1.26 (0.86–1.83)
Extreme dipping				
Crude model	1.04 (0.60–1.79)	1.25 (0.66–2.38)	1.38 (0.68–2.8)	1.58 (0.83–2.99)
Adjusted model^a^	1.02 (0.60–1.74)	1.11 (0.58–2.09)	1.03 (1.01–1.05)	1.03 (1.01–1.04)
Exacerbated systolic morning surge				
Crude model	1.42 (0.85–2.46)	1.34 (0.75–2.55)	1.57 (0.82–3.33)	1.06 (0.62–1.88)
Adjusted model^a^	1.46 (0.82–5.88)	1.35 (0.71–2.60)	1.29 (0.98–1.07)	1.02 (0.99–1.07)
Exacerbated diastolic morning surge				
Crude model	1.82 (1.07–3.25)	3.22 (1.55–7.84)	3.61 (1.57–10.5)	1.66 (0.93–3.16)
Adjusted model^a^	1.78 (1.00–3.16)	3.18 (1.41–7.15)	3.66 (1.43–9.39)	1.61 (0.85–1.02)

BF, body fat; CI, confidence interval; BMI, body mass index; BP, blood pressure; DBP, diastolic blood pressure; OR, odds ratio; SBP, systolic blood pressure; WC, waist circumference; WHR, waist-to-height ratio.

Cut-off points: BMI ≥ 25 kg/m^2^ or ≥ 28 kg/m^2^ for adults and older adults, respectively; WHR ≥ 0.5; WC ≥ 90 cm for men and ≥ 80 cm for women; %BF ≥ 25% in men and ≥32% in women; nocturnal dipping: non-dipping <10% and extreme ≥20%; systolic morning surge ≥23.95 mmHg; diastolic morning surge ≥21.2 mmHg.

ELSA-Brasil, 2012–2014.

^a^ Model adjusted for age, sex, race/colour, schooling, physical activity, binge drinking and use of anti-hypertensives.

Lastly, in the sensitivity analysis, results similar to those shown in Tables 4, 5 were observed after additional adjustment for mean BP. Only the associations of overweight, WC and WHR with 24-hour SBP variability, and between WHR and extreme nocturnal dipping, lost statistical significance (data not shown).

## 4. Discussion

This study aimed to examine for associations between total and central adiposity and ambulatory BP parameters among ELSA-Brasil participants. The results reveal that adiposity indicators were positively associated with BP means and variability, extreme nocturnal dipping and exacerbated morning surge, and the associations were more substantial for indicators of central adiposity than of total adiposity.

### 4.1. Comparison with literature

As far as it was possible to ascertain, this is the first study to investigate the association between a broad set of indicators for adiposity, including %BF and WHR, and BP measured by ABPM and the different parameters derived from that measurement. The relationship between overweight and BP has been well established in the literature using casual BP measurements (3, 4). One possible mechanism is that excess adiposity stimulates activation of the sympathetic nervous system by hyperleptinemia which can result in increase in BP (40). Also, overweight individuals are usually insulin resistant (41), which leads to greater reabsorption of sodium by the kidneys (42) and, consequently, acts to elevate BP (43).

Interestingly, the results of this study suggest that the indicators of central adiposity, especially WHR, were more substantially associated with ambulatory BP than the other indicators. Localised fat in the central region is more involved in the appearance of factors that trigger inflammatory processes (44) and the indicators of central adiposity have been considered better able to predict cardiometabolic risk than other indicators (45). Given the relationship between height and cardiometabolic disorders, it is recommended to use height-adjusted WC, i.e., WHR (46). A previous publication on the ELSA-Brasil population demonstrated that this indicator performed similarly or better than other available anthropometric indices, as well as showing good discriminatory power for cardiometabolic outcomes (47). Other advantages of using WHR include the existence of a single cut-off point, with no distinction between men and women and independent of race or ethnicity, as well as the ability of identify cardiometabolic risk in individuals with normal BMI (48).

Therefore, the results of this study fill a gap in the literature, because it is the first to demonstrate the association between WHR and ambulatory BP. Most studies published to date have used only BMI (5–7, 10) and few have explored using other combined measurements (49, 50). Additionally, the BMI, but also the other adiposity indicators mentioned here involve limitations arising from the process of taking anthropometric measurements, which underlines the importance of using them in combination, for a better assessment of body adiposity (19).

This study also found a positive relationship between the indicators of adiposity and BP variability, especially sleeping BP variability, and those associations held even after adjustment by mean BP in that period. These results are highly significant, because excessive BP variability is considered to be a cardiovascular risk factor, regardless of mean BP levels (51). As already mentioned, overweight tends to raise BP by different mechanisms and, as a result, favours arterial stiffening, which is the prime determinant of BP variability (52). The greater the BP variability, the greater the stress on blood vessels, culminating in endothelial dysfunction (53).

In this study, the indicators for adiposity were also associated positively with extreme nocturnal dipping and exacerbated morning surge, which have also been associated with increased cardiovascular risk, including lesions in target organs, such as left ventricular hypertrophy and carotid intima thickening (25, 26). As exacerbated morning surge can result from extreme nocturnal dipping (54), the interrelationship between these two phenomena has been discussed as regards predicting cardiovascular events (16). Note that morning surge and nocturnal dipping can be considered in a broader sense, together with the standard deviation measure, as components of short-term variability, because they reflect the pattern of BP variation over the 24 h of the day (55). Accordingly, the findings of this study are consistent in that they demonstrate the association between adiposity and BP variability on the basis of different parameters.

In this study, an association was also observed between adiposity and casual BP, which was taken at the participant's place of work, before initiating ABPM, and not in controlled conditions, thus contributing to stronger agreement between two methods in mean BP values. In addition, ABPM makes it possible to evaluate cardiovascular risk markers relating to the BP circadian cycle, such as sleeping BP, nocturnal dipping and morning surge, which cannot be evaluated by other methods of measurement, such as casual or office measurement (13).

### 4.2. Strengths and limitations

The major limitation is cross-sectional design of the study, which does not allow us to establish a cause-effect relationship between exposure and outcome variables. Another limitation is that, in this study, HTN classification by casual BP was based on a one-time measurement (average of two consecutive measurements), compared with 2–3 different visits to confirm sustained elevations in BP in clinical practice (56). However, this procedure is widely used in epidemiological studies, especially those with large sample sizes (57–59). Furthermore, HTN classification was used only in the descriptive analysis and casual BP levels were included as a secondary outcome in our study to compare results with multiple BP readings from ABPM over a 24-hour period.

Main study strengths include the use of data from an internationally cohort (ELSA-Brasil), in which information was collected with great methodological rigour (23). Another strong point is that the waking and sleeping time intervals, which are needed in order to calculate the various ABPM parameters examined, were determined on the basis of times reported by the participants rather than pre-established timetables (24).

### 4.3. Conclusion and future directions

In conclusion, the results of this study reveal that the indicators of adiposity were positively associated with ambulatory BP in the various periods of the day (24-hour, waking and sleeping), including the BP means and variability, extreme nocturnal dipping and exacerbated morning surge, and results were more substantial for indicators of central adiposity than of total adiposity. These findings underline the importance of implementing interventions to control obesity, so as to reduce the frequency of elevated BP and of alterations in the various components of BP. Additionally, they emphasise the importance of using combinations of different anthropometric indicators in primary care, as well as using ABPM in clinical practice and in research. In order to better understand the relationship between body adiposity and ABPM parameters, longitudinal studies could be used in future research.

## Data Availability

The original contributions presented in the study are included in the article, further inquiries can be directed to the corresponding author.
